# Comparison of antibiotic prescribing between physicians and advanced practice clinicians

**DOI:** 10.1017/ice.2023.175

**Published:** 2024-01

**Authors:** Adam L. Hersh, Daniel J. Shapiro, Guillermo V. Sanchez, Lauri A. Hicks

**Affiliations:** 1 Division of Infectious Diseases, Department of Pediatrics, University of Utah, Salt Lake City, Utah; 2 Division of Pediatric Emergency Medicine, Department of Pediatrics, Boston Children’s Hospital, Boston, Massachusetts; 3 Office of Antibiotic Stewardship, Division of Healthcare Quality Promotion, National Center for Emerging Zoonotic and Infectious Diseases, Centers for Disease Control and Prevention, Atlanta, Georgia

## Abstract

We compared antibiotic prescribing rates for respiratory conditions in a national sample of outpatient visits from 2010 to 2018 between physicians and advanced practice clinicians (APCs). APCs prescribed antibiotics more frequently than physicians (58% vs 52%), but there were no differences in selection of guideline recommended first-line agents between specialties.

Inappropriate use of antibiotics in outpatient care is common and harmful to patients. Since the Centers for Disease Control and Prevention (CDC) developed the Core Elements of Outpatient Antibiotic Stewardship, modest reductions in inappropriate antibiotic prescribing have occurred nationwide.^1,2^ However, substantial variation in prescribing rates exist across geographic regions, clinical settings, and clinician specialties.^1,2^

Advanced practice clinicians (APCs) include nurse practitioners and physician assistants, and APCs comprise a growing share of the clinician workforce and of antibiotic prescriptions.^3^ APCs accounted for >30% of antibiotic prescriptions nationwide in 2020, compared to only 14% in 2011.^4^ Prior studies indicated that APCs prescribe antibiotics more frequently than physicians for patients with similar diagnoses. However, little is known about whether differences in antibiotic selection practices for first-line agents exist between clinician groups.^5–8^ We compared national rates of antibiotic prescribing and appropriate antibiotic selection between physicians and APCs for common respiratory conditions.

## Methods

In this retrospective study of ambulatory care visits for acute respiratory tract infections (ARTIs), we used data from the National Ambulatory Medical Care Survey and the National Hospital Ambulatory Medical Care Survey from 2010 to 2018, excluding 2017 because data from all settings were not available for that year. These surveys are administered annually by the National Center for Health Statistics and employ a complex probability sampling design to allow for nationally representative estimates of care provided at office and emergency department visits in the United States. Survey elements include clinician characteristics, patient demographics, diagnoses, and medications prescribed.^4^

The primary outcomes were (1) the proportion of visits in which antibiotics were prescribed for ARTIs and (2) the proportion of visits for acute otitis media (AOM), sinusitis, or pharyngitis in which appropriate first-line antibiotics were selected. We defined ARTIs using *International Classification of Diseases, Ninth* and *Tenth Revision* (ICD-9 and ICD-10) codes according to a previously published scheme.^2^ Among ARTIs, we further characterized the diagnosis according to whether antibiotics were almost always indicated (tier 1), sometimes indicated (tier 2), or rarely indicated (tier 3). Definitions for antibiotics and appropriate first-line antibiotics were defined as amoxicillin or amoxicillin-clavulanate for AOM and sinusitis and amoxicillin or penicillin for pharyngitis.^9^ Outcomes were compared between visits in which APCs provided care and those in which physicians provided care. APCs were defined as nurse practitioners or physician assistants according to a checkbox on the survey instrument that indicated the types of clinicians present at the encounter.

Proportions were compared between groups using a χ^2^ test, and time trends were assessed using logistic regression with year as the predictor variable. To assess differences in case mix among patients seen by APCs versus physicians, we compared the proportion of visits involving patients with a chronic condition, which were defined as obesity, hypertension, hyperlipidemia, coronary artery disease, congestive heart failure, chronic obstructive pulmonary disease, cerebrovascular disease, cancer, asthma, end-stage renal disease, or diabetes. These diagnoses were established according to check boxes on the survey form. To assess independent associations between clinician type and each of the primary outcomes, we performed multivariable logistic regression models controlling for patient age, sex, race, ethnicity, US Census Region, metropolitan statistical area, insurance status (private, public, and self-pay or other), setting (office vs emergency department), diagnosis, clinician specialty, and year. All analyses were performed using Stata version 14 software (Stata Corp, College Station, TX) and accounted for all aspects of the complex probability sampling design including primary sampling units, strata, and visit weights.

## Results

Based on an unweighted sample of 43,935 visits, there were an estimated 95,331,202 visits per year for ARTIs in ambulatory care settings, 87% of which were to offices. Overall, 11% of these visits included an APC, and 45% were for diagnoses for tier 3 diagnoses. The percentage of visits that included an APC between 2010 and 2018 was stable (*P* = .55). There was no difference in the presence of chronic conditions among visits involving APCs and those involving physicians (33% vs 34%; *P* = .70).

The proportion of visits in which antibiotics were prescribed was higher for APCs (58%) than for physicians (52%; *P* = .01), and this difference was stable across years. The difference in antibiotic prescribing was most pronounced in the subgroups of visits among children (56% vs 49%; *P* = .02), in offices (60% vs 52%; *P* = .03), and for tier 2 diagnoses (72% vs 66%; *P* < .01) (Table 1). In the analysis of antibiotic selection for tier 2 diagnoses, appropriate first-line antibiotics were prescribed in 49% of visits, and there was no difference in selection of first-line antibiotics between APCs and physicians (Table 2).

**Table 1.** Proportion of Respiratory Visits with Antibiotics Prescribed, Stratified by Clinician Type, 2010–2018, Excluding 2017

Subpopulation	Physician %	APC %	Overall %	*P* Value (χ^2^)
All patients	52	58	53	.01
**Age group**				
Children	49	56	50	.02
Adults	55	60	56	.14
**Setting**				
Offices	52	60	53	.03
EDs	53	55	53	.11
**Diagnosis**				
Tier 1	62	66	63	.55
Tier 2	66	72	67	.01
Tier 3	36	41	37	.14

Note. APC, advanced practice clinician; ED, emergency department; tier 1, antibiotics were almost always indicated; tier 2, antibiotics sometimes indicated; tier 3, antibiotics rarely indicated.

Source: National Ambulatory Medical Care Survey and the National Hospital Ambulatory Medical Care Survey, United States.

**Table 2.** Proportion of Visits in which First-line Antibiotics Were Prescribed for Tier 2 Diagnoses, Stratified by Clinician Type, 2010–2018, Excluding 2017

Subpopulation	Physician %	APC %	Overall %	*P* Value (χ^2^)
AOM	56	56	56	.83
Sinusitis	44	40	44	.56
Pharyngitis/tonsillitis	47	52	48	.28
Combined	49	49	49	.91

Note. APC, advanced practice clinician; AOM, acute otitis media.

Source: US National Ambulatory Medical Care Survey and US National Hospital Ambulatory Medical Care Survey.

In our multivariable logistic regression analysis controlling for patient and clinician characteristics, the odds of an antibiotic prescription were higher during visits with APCs than with physicians (adjusted odds ratio [aOR], 1.3; 95% CI, 1.1–1.7). Higher odds of prescribing during APC visits extended to tier 2 diagnoses (aOR, 1.2; 95% CI, 1.0–1.6) and tier 3 diagnoses (aOR, 1.4; 95% CI, 1.0–2.0) when examined independently. There was no association between clinician type and selection of first-line antibiotics for tier 2 diagnoses (aOR, 1.0; 95% CI, 0.7–1.2).

## Discussion

In this nationally representative sample of outpatient visits involving physicians and APCs for respiratory conditions, the odds of an antibiotic prescription were 30% higher when APCs were present during the visit. There were no differences between physician-only and APC-associated visits in the use of first-line antibiotics for AOM, pharyngitis, and sinusitis.

Higher antibiotic prescribing during APC-associated visits compared to physician-only visits has been shown in previous studies.^5–8^ The reasons for this difference are unclear and likely multifactorial. Although case-mix differences between patients seen by these clinician groups could be a contributing factor, we observed no differences in the prevalence of comorbidities, and the higher prescribing rate remained even when diagnoses were stratified by tiers and adjusted in multivariable modeling. Differences in educational emphasis on antibiotic stewardship in medical training or continuing education opportunities between specialties may play a role. Many physician professional organizations have emphasized stewardship, and stewardship leaders are typically physicians and not APCs.

There was no difference in use of appropriate first-line antibiotics for common conditions, ∼50% for each and well below national targets.^9^ This finding suggests that efforts around education and impact of clinical guidelines for antibiotic selection require substantial enhancement for all clinician specialties.

This study had several limitations. Although we examined subgroups of respiratory encounters by tier and found no difference in comorbidities, case-mix differences between patients seen by physicians versus APCs may remain. We were unable to account for antibiotic allergies. The sampling frame used by these data sets does not encompass all practice settings, such as urgent care and retail clinics; thus, prescribing patterns in these settings may be different between specialties than in physician offices. Additionally, the sampling approach is based on selecting for physician practices, which may exclude settings where APCs practice without physicians present.

Due to increasing contributions of APCs to outpatient care and antibiotic prescribing, antibiotic stewardship interventions should better incorporate APCs alongside all clinician specialties. The CDC is actively engaging with APC professional societies to incorporate stewardship into professional meetings and to tailor education materials for these audiences. Additional opportunities exist within health systems to support APC participation in stewardship program development and leadership.
